# Transcriptomic analysis revealed potential regulatory biomarkers and repurposable drugs for breast cancer treatment

**DOI:** 10.1002/cnr2.2009

**Published:** 2024-05-08

**Authors:** Most Shornale Akter, Md. Helal Uddin, Sheikh Atikur Rahman, Md. Arju Hossain, Md. Ashiqur Rahman Ashik, Nurun Nesa Zaman, Omar Faruk, Md. Sanwar Hossain, Anzana Parvin, Md Habibur Rahman

**Affiliations:** ^1^ Department of Biotechnology and Genetic Engineering Islamic University Kushtia Bangladesh; ^2^ Department of Biotechnology and Genetic Engineering Mawlana Bhashani Science and Technology University Tangail Bangladesh; ^3^ Department of Microbiology Primeasia University Dhaka Bangladesh; ^4^ Department of Statistics Jagannath University Dhaka Bangladesh; ^5^ Department of Computer Science and Engineering Islamic University Kushtia Bangladesh; ^6^ Center for Advanced Bioinformatics and Artificial Intelligence Research Islamic University Kushtia Bangladesh

**Keywords:** biomarker, breast cancer, hub‐protein, in silico analysis, molecular docking, transcriptomics

## Abstract

Breast cancer (BC) is the most widespread cancer worldwide. Over 2 million new cases of BC were identified in 2020 alone. Despite previous studies, the lack of specific biomarkers and signaling pathways implicated in BC impedes the development of potential therapeutic strategies. We employed several RNAseq datasets to extract differentially expressed genes (DEGs) based on the intersection of all datasets, followed by protein–protein interaction network construction. Using the shared DEGs, we also identified significant gene ontology (GO) and KEGG pathways to understand the signaling pathways involved in BC development. A molecular docking simulation was performed to explore potential interactions between proteins and drugs. The intersection of the four datasets resulted in 146 DEGs common, including AURKB, PLK1, TTK, UBE2C, CDCA8, KIF15, and CDC45 that are significant hub‐proteins associated with breastcancer development. These genes are crucial in complement activation, mitotic cytokinesis, aging, and cancer development. We identified key microRNAs (i.e., hsa‐miR‐16‐5p, hsa‐miR‐1‐3p, hsa‐miR‐147a, hsa‐miR‐195‐5p, and hsa‐miR‐155‐5p) that are associated with aggressive tumor behavior and poor clinical outcomes in BC. Notable transcription factors (TFs) were FOXC1, GATA2, FOXL1, ZNF24 and NR2F6. These biomarkers are involved in regulating cancer cell proliferation, invasion, and migration. Finally, molecular docking suggested Hesperidin, 2‐amino‐isoxazolopyridines, and NMS‐P715 as potential lead compounds against BC progression. We believe that these findings will provide important insight into the BC progression as well as potential biomarkers and drug candidates for therapeutic development.

## 1 INTRODUCTION

Breast cancer (BC) is an aggressive form of cancer that develops in the breast cells, marked by uncontrollable cell growth, resulting in a lump or tumor. Based on fatality rates, breast cancer is the second most prevalent cancer, and at the molecular level, it is heterogeneous.^1^ About 70%–80% of patients are curable during the non‐metastasis stages, known as the early stages. BC has an extremely low overall survival rate of 91% during 5 years, with the correct diagnosis and treatment during the primary stage, there is a good possibility of recovery.^2^ Globally, around 2.3 million women were diagnosed and 685 000 people died of BC. Furthermore, the report indicates a new case being identified every 18 s.^3^ In 2020, according to GLOBOCAN, 13028 new breast cancer cases were reported in Bangladesh, the most common cancer form among women at 19%. On a global scale the number of new cases and deaths from breast cancer will rise by 2040, particularly in Asia and Bangladesh.^4^

For BC progression, there are typically two categories of risk factors: causative and non‐causal. It is thought that defective genes are the underlying risk factors for BC. Mutation of estrogen and progesterone are major causal factors involved in the development of BC.^5^ Non‐causal factors include some epigenetic factors, drinking alcohol, body mass index (BMI), height, density of breast tissue in mammograms, age of first menstruation, age of onset of menopause, level of physical activity, smoking, and having type 2 diabetes that increase the likelihood of developing breast cancer.^6^, ^7^ About 30 genes are associated with the risk of breast cancer. These encompass high‐risk early‐onset breast cancer genes like BRCA1 and BRCA2, along with various rare cancer syndrome genes and less potent rare genes.^8^ Understanding the molecular mechanisms and pathogenesis processes of BC requires the discovery of both causal and non‐causal genetic risk factors.

One of the most widely used approaches to pinpointing the hub gene that contributes to disease is transcriptomic data analysis. Several studies have been conducted and published in the last few years using public databases such as GREIN and Gene Expression Omnibus (GEO) to predict potential biomarkers. Liu et al. conducted a study of 1203 BC samples from The Cancer Genome Atlas Database and identified 1317 differentially expressed genes, with 744 genes showing upregulation and 573 genes showing downregulation.^9^ Besides, Wang et al. also used one data set (GSE45827) from the GEO database and identified distinct expression genes in BC.^10^ Based on the examination of single transcriptome datasets, multiple studies have predicted different sets of hub genes for BC,^11^, ^12^, ^13^ but none of them shared a single hub gene. Researchers typically integrate numerous datasets generated in different situations to uncover more reliable DEGs between case and control samples. Multiple transcriptome datasets have also been investigated by specific research to identify more prevalent and stable hub genes that cause BC.^14^, ^15^, ^16^ As a result, for this analysis, we collected 5 transcriptomic datasets from different regions to identify potential shared hub genes, regulatory biomolecules and repurposed drugs for BC.

**FIGURE 1:**
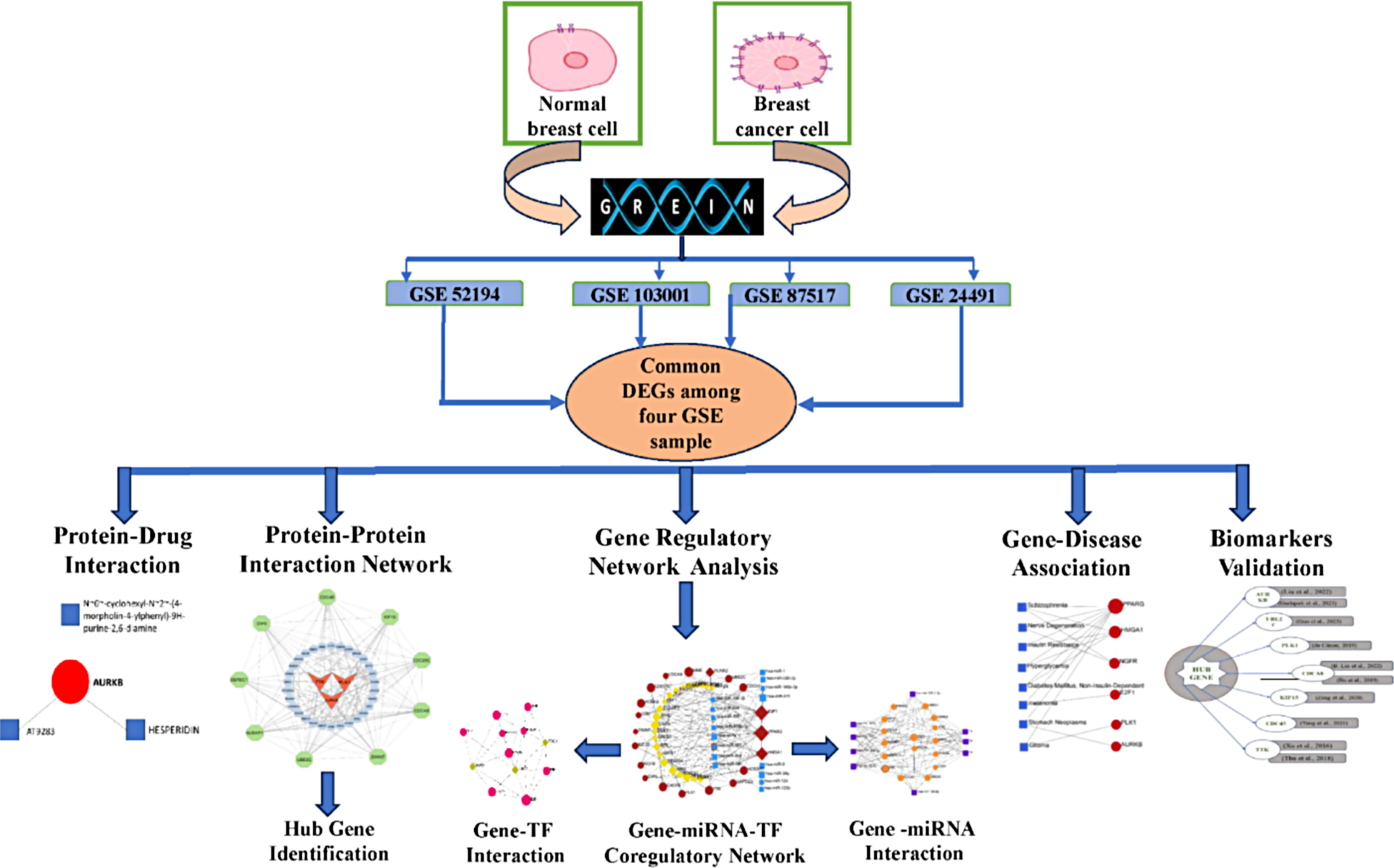
Methodology of our proposed study. The Gene Expression (GEO) datasets were evaluated to discover shared differentially expressed genes (DEGs). Enrichment analysis was used to identify significant signaling pathways and Gene Ontology (GO) concepts. The network of protein–protein interactions was examined to find hub proteins. Regulatory macromolecules such as transcription factors (TF) and micro RNA were identified. We have also evaluated protein‐drug candidate compounds and gene‐disease association, and finally, molecular docking validated our identified hub proteins.

Drug repurposing (DR) is a potential method to address many of the challenges in discovering and developing new therapeutic candidates, based on the novel clinical implications of currently existing FDA‐approved medications that were developed for a variety of conditions.^17^ Genomic markers‐induced proteins are crucial receptors, and transcriptomic analysis is a prominent genomic biomarker identification method. Several researchers predicted genomic biomarkers to study the molecular mechanisms and pathogenesis processes of BC.^18^, ^19^ In addition, for the treatment of BC, some of them proposed potential drug candidates.^20^, ^21^ The data they released did not show a common set of receptors or medications, and none of them have tested their indicated pharmaceuticals against independent receptors proposed by others via molecular docking. This project aims to computationally identify shared genomic biomarkers (drug targets) for BC and highlight their roles, pathways, and regulatory molecules like transcription factors and miRNA, as well as explore genomic biomarker‐guided candidate drugs for BC treatment. Then, molecular docking was utilized to confirm strong affinity and higher interaction between the candidate drugs and potential hub‐targets (biomarkers). Figure 1 represented the flow diagram of our proposed study.

## 2 MATERIALS AND METHODS

### 2.1 Recognition of data sources and statistical analysis of DEGs

We collected RNA‐seq data from the GREIN website (http://www.ilincs.org/apps/grein/?gse).^22^ We focused on datasets that exhibit distinct case and control groups. These datasets contain a minimum of 5 cases and 5 controls, along with specific criteria that aid in our analysis. We utilized four GSE datasets: GSE103001, GSE87517, GSE24491, and GSE52194.

The GREIN servers were also utilized to identify differentially expressed genes in transcriptomic analysis. The selected datasets focused on those with a high representation of genes associated with breast cancer.^22^ Then, Statistical operations, including moderate t‐statistics, B‐statistics, and ANOVA test for all the pair‐wise comparisons, were performed on the datasets to determine and verify the DEGs via Pomelo II (http://pomelo2.bioinfo.cnio.es) web server.^23^ Additionally, the Benjamini‐Hochberg false discovery rate (FDR) of <.05 approach was employed to balance the discovery of statistically significant genes and the limitation of false positives.^24^ In this study, genes with absolute *p*‐value <.05 and absolute log2 fold‐change >1 were considered for DEGs. We also regarded as log2 fold‐change ≥1 and log2 fold‐change ≤ −1 criteria to explore up and down‐regulated genes, respectively.^25^

Using an online tool called jVenn (jvenn inra.fr), we discovered common genes that are shared among four different datasets.^26^ We determined the datasets that provided the highest number of common genes, which we will use for more robust analysis. jVenn not only provided a list of these shared genes but also generated visual representations of the overlaps.^26^

### 2.2 Protein–protein interaction analysis and detection of hub protein

We analyzed protein–protein interactions of the shared DEGs using the STRING (https://string-db.org/) database through the Network Analyst web server.^27^ The PPI network was constructed using the generic PPI option, focusing on *H. sapiens* as the organism. To identify effective hub proteins in the PPI network, we employed various methods within the Cytoscape software through the cytoHubba plugin.^28^ Local methods rank hubs based on their relationships with neighboring nodes, while global methods consider node relationships within the entire network. For the identification of unique hub genes, we utilized five different methods of Cytoscape, including maximum neighborhood component (MNC), Degree, and MCC (maximum clique centrality) in the local network, while Closeness, and Bottleneck algorithms in the global network. Local methods rank establishing based on their relationships with neighboring nodes, while global methods consider node relationships within the entire network.^29^ By comparing and evaluating the data, we pinpointed common nodes or hubs with the highest significance. Finally, for customization, we employed Cytoscape v3.7 to visualize our personalized networks.

### 2.3 Gene ontology with pathway analysis

GO (Gene Ontology) and KEGG (Kyoto Encyclopedia of Genes and Genomes) are widely used methods for identifying significantly enriched functions through pathway annotation. This involves categorizing biological processes (BP), molecular functions (MF), and cellular components (CC), as well as pathways related to select Differentially Expressed Genes (DEGs). Biological processes encompass the sequence of changes that occur as cells progress through various stages, often involving one or multiple genes to achieve diverse biological objectives. Molecular functions pertain to the biochemical roles of gene products, while cellular components refer to the specific locations within a cell where gene products function. KEGG offers validated pathways insights into drug development, human disorders, cellular processes, and organismal systems.

In this analysis, a significance threshold is set at a *p*‐value below .05, indicating the statistical relevance of functional enrichment. Commonly used website tools like DAVID and SRplot are employed for the analysis. This process reveals significant and enriched terms that hold valuable implications for practical outcomes.

### 2.4 Regulatory biomolecules identification

Some regulatory molecules, such as microRNAs (miRNAs) and transcription factors (TFs) play a crucial role in altering gene expression outcomes and controlling transcription processes.^29^ In the realm of molecular, biological, and cellular processes, gene transcriptional regulations hold significant importance. Gene regulatory networks govern the levels of mRNA and protein expression. Transcription factors (TFs), which are proteins, exert influence over transcription by binding to specific DNA regions, making them key players in these networks. In the human genome, approximately 1600 TFs have been identified. MicroRNAs (miRNAs), on the other hand, are non‐coding RNA molecules that participate in RNA silencing and post‐transcriptional regulation. Roughly 1900 miRNAs have been identified in the human genome. The interactions between TFs and hub proteins create an undirected graph, where TFs are represented as nodes, and their interactions with hub proteins are depicted as edges. The top hub‐TF refers to the TF node with the highest number of interactions with hubs. JASPAR is used to determine TFs‐HubGs interactions.^30^ Utilizing NetworkAnalyst(https://www.networkanalyst.ca/), we can pinpoint key miRNAs that govern hub proteins.^31^ This involves scrutinizing the interactions between miRNAs and hub proteins in the TarBase and miRTarBase databases.^31^, ^32^ The identification of top miRNAs is based on their highest topology. To ensure reliable outcomes, the process can be repeated using EnrichR and miRTarBase databases within the JASPAR framework.^33^

### 2.5 Gene‐disease association analysis

A Gene Disease association is a type of analysis which is used in bioinformatics to understand the complex interactions between phenotype–genotype relationships and the mechanisms underlying genes and diseases.^34^ Here, we used our unique hub gene to analysis the gene‐disease association in the DisGeNET database through the support of NetworkAnalyst (https://www.networkanalyst.ca/) online website.^26^

### 2.6 Protein drug interaction

Protein‐drug interaction shows the network between the protein and the drug. Through this interaction, we can easily select the drug as an inhibitor of specific proteins.^35^ So, for identification of the drug molecule for our three common hub proteins, we used the NetworkAnalyst online website on the DrugBank database (https://www.networkanalyst.ca/) and found some drug molecules that we can use as inhibitors of our target proteins.

### 2.7 Candidate drug prediction through molecular docking

We used molecular docking to find FDA approved drug that has been validated in silico for use against breast cancer. This involves analyzing the interaction between drug agents and receptor proteins, where our central hub proteins act as the receptors. RCSB Protein Data Bank (https://www.rcsb.org/search) was utilized to extract the 3D structures of the respective PDB ID (4af3, 2x9e, and 1q4k) against target proteins AURKB, TTK and PLK1.^36^ To enhance the molecular docking capabilities of the receptor proteins, any pre‐bound ligands and water molecules were eliminated, and polar hydrogen atoms were added through Biovia Discovery Studio Visualizer‐2021 Client. Finally, the negative energy of each protein 3D structure was calculated using the GROMACS 43B1 force field of SwissPDB viewer software.^37^

To carry out molecular docking, we have also required three‐dimensional structures of candidate drugs. The SDF format of the top three FDA‐approved pharmacological agents of AURKB and five approved drugs of PLK2 and one approved drug of TTK proteins were also downloaded from the PubChem database (https://pubchem.ncbi.nlm.nih.gov/).^38^ Then Open babel software was used to convert the 2D SDF to the 3D SDF structure of each drug candidate.^39^ The ligand structures were initially imported into PyRx software^40^ in 3D SDF format using the open babel tool that is also built into PyRx. The universal force field was optimized by adding charges in order to achieve minimum energy consumption. After adding charges and adjusting the universal force field to allow for energy minimization, the ligands were then converted to AutoDock Ligand format (pdbqt).

PyRx with Vina Wizard was used in molecular docking experiments to determine binding affinity and ligand‐receptor interactions responsible for anticancer, antioxidant and phytotoxic activities.^40^ By utilizing the Vina Wizard Control in the PyRx software, the protein and multiple ligands to be bound were chosen.^41^ For the grid box used in the docking performance, the coordinates center_x:y:z: = −15.6205: −16.6734: −3.6226; and size_x:y:z = 47.1779: 57.7232: 60.5246 were supplied. Receptor protein‐key active phytochemicals binding interactions were visualized using the Discovery Studio program. The docking results and root‐mean‐square deviation (RMSD) values of the most promising conformations were chosen for in‐depth analysis.

### 2.8 Validation of potential biomarkers

To validate our identified biomarkers, including hub proteins, transcription factors and micro‐RNAs, we have vigorously searched the literature studies or related publications that are strongly connected to disease development. Then, the findings were represented in a figure with proper citations. By settings relapse‐free survival at 5 years (*n* = 1329), and chemotherapy option in breast cancer patient data, we have employed ROC Plotter website (https://www.rocplot.org/) to validate three hub proteins (AURKB, PLK1 and TTK) by ROC curve analysis. ROC analysis provides a comprehensive evaluation of sensitivity and specificity across different thresholds. The area under the ROC curve (AUC) is a valuable metric, with higher AUC values indicating better discriminatory power. A detailed ROC analysis helps in setting an optimal threshold for biomarker performance.^42^

## 3 RESULTS

### 3.1 Selection of differential gene expression (DEGs)

**FIGURE 2:**
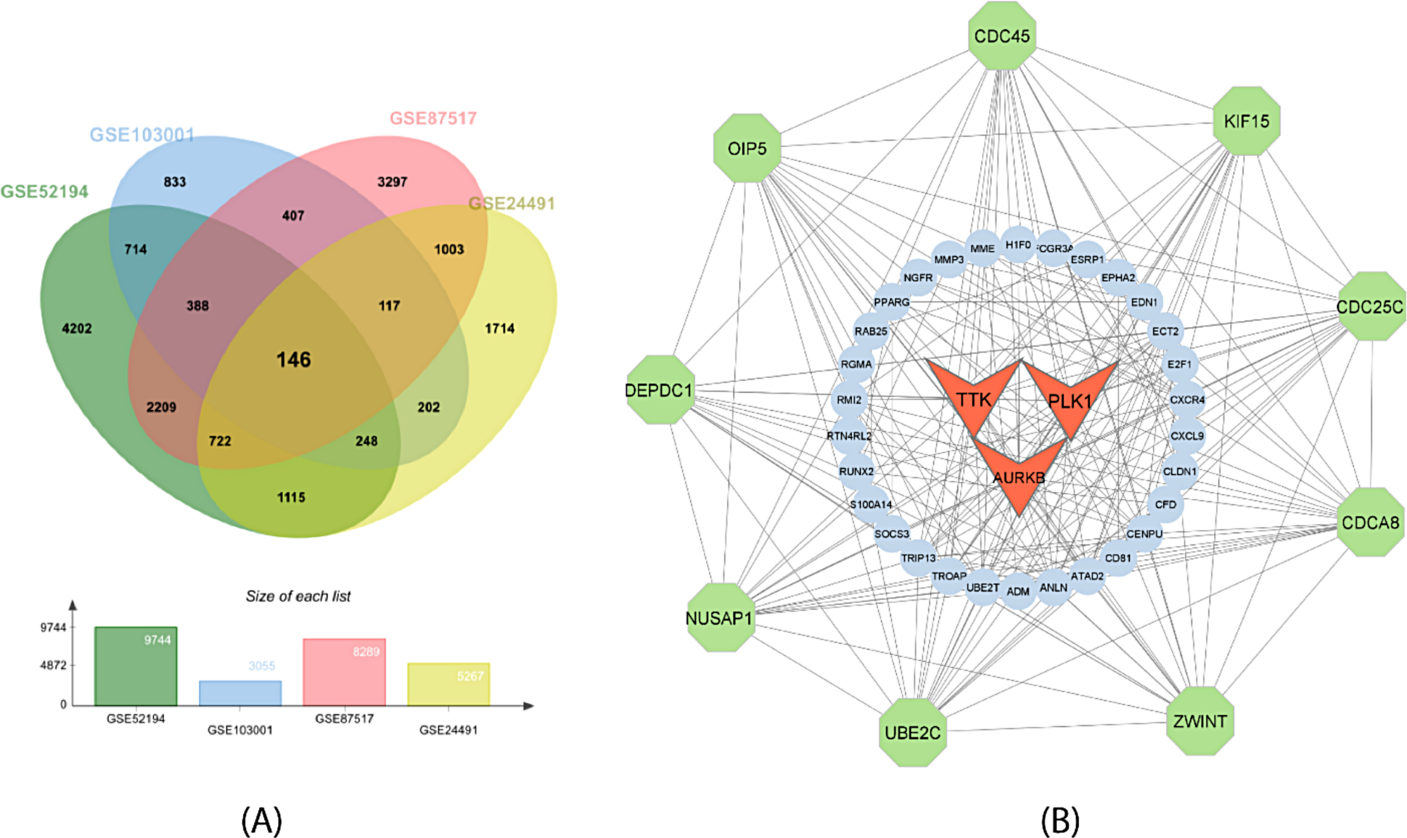
Common significant differentially expressed genes (DEGs) identified from four selected Gene Expression Omnibus (GEO) database‐based datasets. (B) Protein–protein interaction network provided edge number 240 and seed number 48 identified from the Cytoscape. Red‐colored and triangle‐shaped represent three genes with the most significant interactions, while outer greeney‐colored and octagonal‐shaped represent nine genes with a significant gene that interacts with many other genes.

RNA‐seq datasets that give DEGs were selected from the GREIN web server. Identified datasets contain a large number of breast cancer genes. Here, the cutoff range of absolute logFC>1 and absolute *p*‐value <.05 is set up to find DEGs; for up‐regulated gene, log FC ≥1, and for down‐regulated gene, log FC≤ −1, with *p*‐value <.05 were also considered. We selected 4 datasets, including GSE103001, GSE87517, GSE24491, and GSE52194, having a total of 10 371, 3170, 8884, and 5599 genes. Here, 4329, 1470, 3572, and 3103 were upregulated genes and 6042, 1700, 5312, and 2494 were downregulated genes identified. All over 4 datasets, we found 146 common DEGs, which is the highest number in our analysis and presented in Venn diagram (Figure 2A). Besides, details of collected datasets and statistical analysis result were provided in Table 1.

**TABLE 1:** Details of collected datasets with data sources, experiment types, and number of differentially expressed genes.

GEO Accession No	Source of sample	Experiment type	Total DEGs	Upregulated DEGs	Down‐regulated DEGs
1. GSE52194	Breast; Breast cancer tissue	Using mRNA sequencing	10 371	4329	6042
2. GSE103001	Mammary; tumor tissue	Stranded RNA sequencing	3170	1470	1700
3. GSE87517	Breast; Tumor tissue	In situ	8884	3572	5312
4. GSE24491	Breast; Breast cancer tissue	High throughput SAGE sequencing	5597	3103	2494

Abbreviations: DEGs, differentially expressed genes; GEO, gene expression omnibus; SAGE, serial analysis of gene expression.

### 3.2 Detection of hub protein through PPI analysis

**FIGURE 3:**
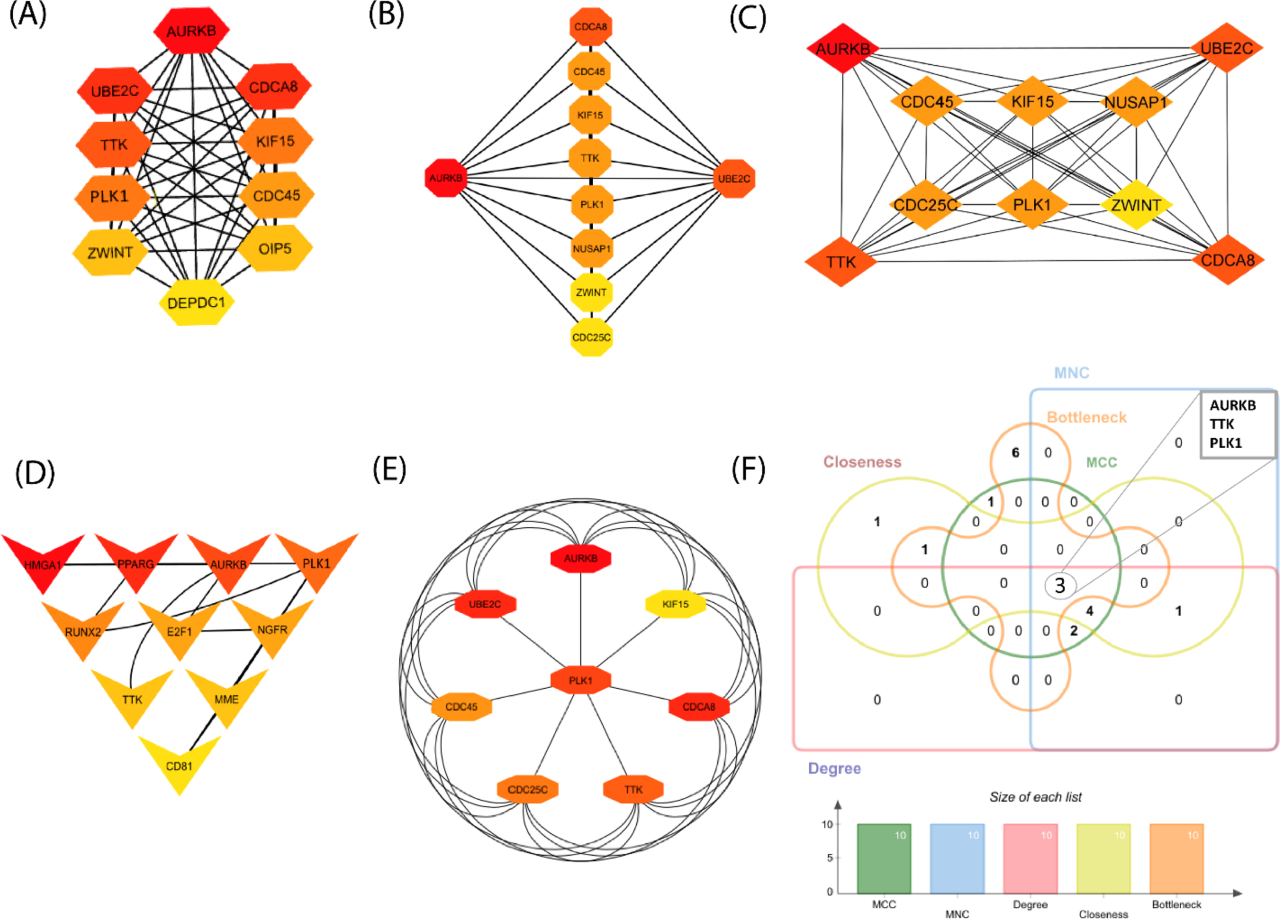
Several CytoHubba algorithms including (A) MCC, (B) MNC, (C) Degree, (D) Closeness and (E) Bottleneck were used to determine the hub gene from the PPI network. Red to Yellow color gradients suggest that hub genes are more highly to lower ranked. (F) jVenn diagram represented common 3 hub proteins among 5 cytoHubba methods of Cytoscape.

Protein–Protein interaction (PPI) networks constitute a crucial field of study, offering insights into the interactions among cellular proteins. Utilizing the STRING database, which employs a cutoff score of 900 to establish general PPI interactions, we uncovered protein–protein interaction (PPI) networks involving the chosen DEGs. Our PPI network encompasses edge 240 and seed 48 from the highest‐ranking 12 proteins including AURKB, TTK, PLK1, NUSAP1, UBE2C, ZWINT, CDCA8, CDC25C, KIF15, CDC45, OIP5 and DEPDC1 visually represented in PPI interactions (Figure 2B). These selected proteins are linked to processes such as ubiquitin‐mediated proteolysis, osteoclast differentiation, apoptosis, focal adhesion, and homologous recombination. We also employed Cytoscape software to visualize PPI interactions, aiming to identify the most interconnected hub proteins among the DEGs. Cytoscape incorporates CytoHubba, which employed techniques such as Degree, MNC, Closeness, MCC and Bottleneck to predict the top 10 hub proteins given in Figure 3A–E. In this study, we found 3 common DEGs involved in all five methods, including AURKB, TTK, and PLK1 shown in Figure 3F. These chosen hub proteins have potential as biomarkers for early‐stage breast cancer for prognosis investigations.

### 3.3 Functional enrichment and pathway analysis

**FIGURE 4:**
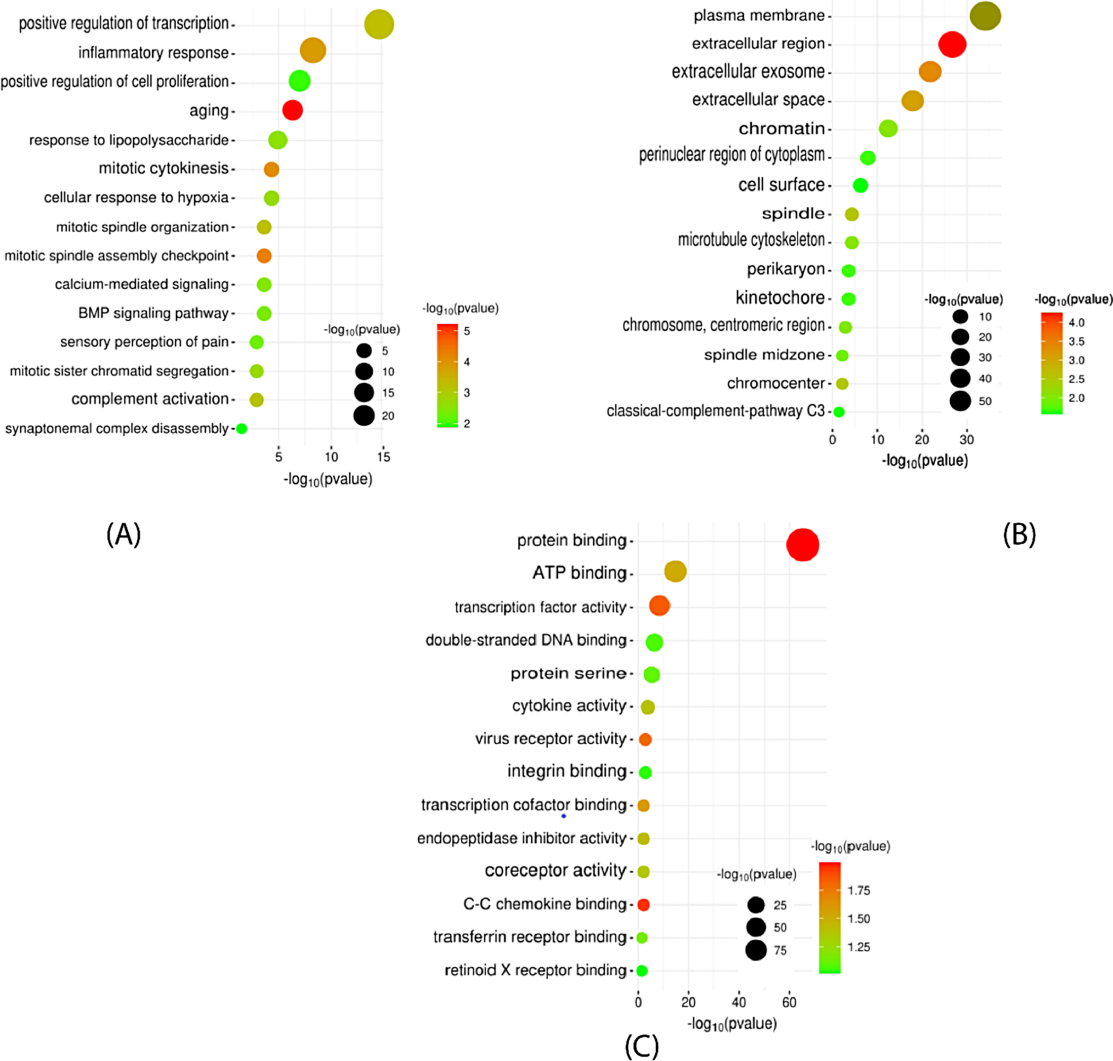
A bubble plot is utilized for gene ontology (GO) pathway analysis based on the −log_10_(p)value. (A) Biological Process, (B)Cellular Components, and (C) Molecular Function. Larger bubbles signify a higher number of genes associated with a particular process or pathway, while smaller bubbles indicate fewer genes involved. The colors of the bubble plots correspond to the −log_10_(P‐value) of the respective genes.

**FIGURE 5:**
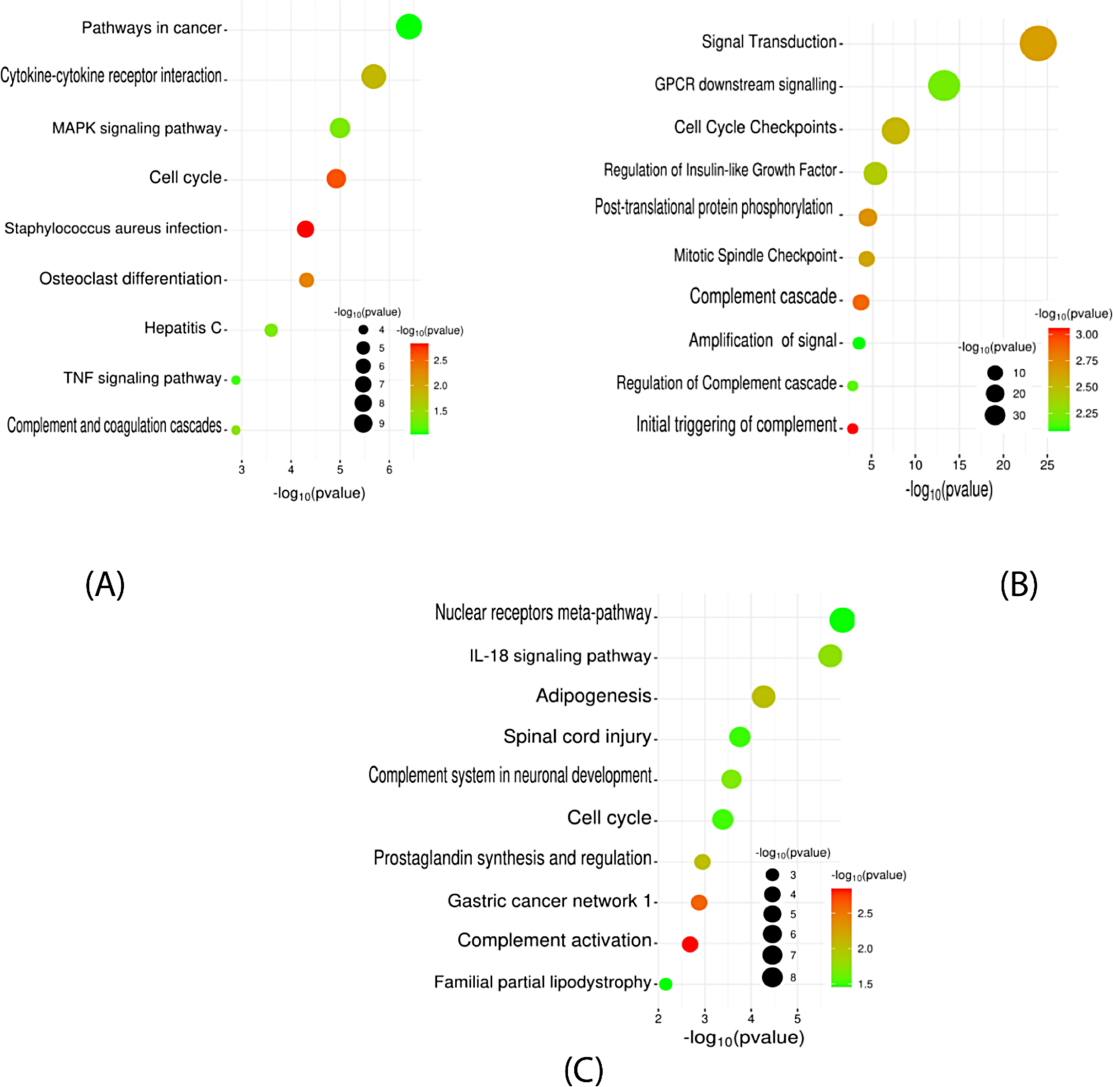
A bubble plot is utilized for gene ontology (GO) pathway analysis based on the −log_10_(p)value. (A) KEGG pathways, (B) Reactome pathways, and (C) WiKi pathways. Larger bubbles signify a higher number of genes associated with a particular process or pathway, while smaller bubbles indicate fewer genes involved. The colors of the bubble plots correspond to the −log_10_(P‐value) of the respective genes.

The aim is to identify the signaling pathways and pertinent Gene Ontology (GO) terms that show significant enrichment with the DEGs in breast cancer. The GO terms comprise molecular functions (MF), which involve protein binding, ATP binding, transcription factor activation, double‐stranded DNA binding, and protein serine, etc. The biological process (BP) involved are positive regulation of transcription, inflammatory response, positive regulation of cell proliferation, aging, and response to lipopolysaccharide etc. Finally, cellular components (CC) are involved the plasma membrane, extracellular region, extracellular exosome, extracellular space, and chromatin etc. represented in Figure 4A–C. By utilizing SRplot and the DAVID database, functional gene sets of DEGs were enriched using KEGG, Reactome, and WIKI pathways. KEGG pathway involves pathways in cancer, cytokine‐cytokine receptor interaction, MAPK signaling pathway, and cell cycle etc. Reactome pathways were included in signal transduction, GPCR downstream signaling, Cell Cycle Checkpoint, and Regulation of Insulin‐like Growth Factor, etc. In addition, the WIKI pathway revealed in nuclear receptors meta‐pathway, IL‐18 signaling pathway, Adipogenesis, and Spinal cord injury, etc., represented in Figure 5A–C.

### 3.4 Identification of transcriptomic signatures through NetworkAnalyst

**FIGURE 6:**
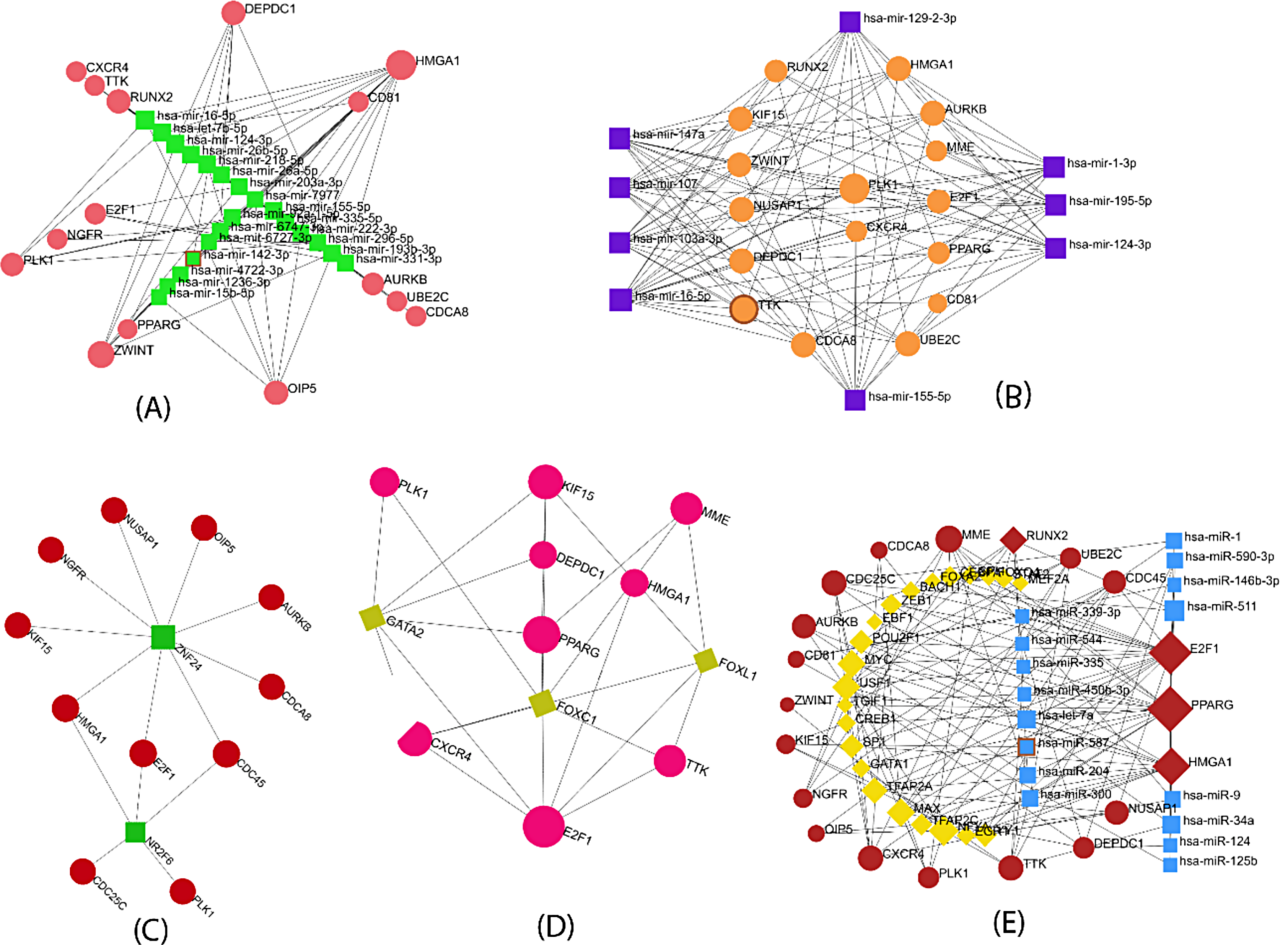
Gene regulatory networks of Breast Cancer. DEGs‐miRNAs network from the (A) miRTarbase database and (B) Tarbase database. Here, a square‐shaped represents miRNA and a round shape represents DEGs. On the other hand, the DEGs‐TFs interaction network from the (C) ENCODE database and (D) Jasper database. Here, the TF represented by diamond‐shaped and circular‐shaped represents differentially expressed genes. (E) DEGs‐TF‐miRNA coregulatory network. Here, square‐shaped and blue colors represent miRNA; diamond‐shaped and yellow colors represent TFs; round‐shaped and maroon colors represent DEGs.

Utilizing miRTarbase from the NetworkAnalyst web server, we found the top 36 miRNAs among them top 5 mi‐RNAs are hsa‐mir‐16‐5p, hsa‐let‐7b‐5p, hsa‐mir‐124‐3p, hsa‐mir‐218‐5p and hsa‐mir‐335‐5p (Figure 6A). By using the Tarbase database from the NetworkAnalyst web server, we found 25 different miRNA connected with 15 hub genes, among them hsa‐mir‐16‐5p, hsa‐mir‐1‐3p and hsa‐mir‐147a hold the top position **(**Figure 6B). To identify transcription factor (TF), we used two methods Encode and Jasper from the NetworkAnalyst web server. In Encode, we found two top TF such as ZNF24 and NR2F6 (Figure 6C **)** and Jasper indicates the top three TFs such as FOXC1, GATA2 and FOXL1 (Figure 6D).

Gene‐miRNA‐TF coregulatory interaction is represented in Figure 6E. We found a total 58 miRNA and 22 transcription factors that connected with 20 hub genes. Among them top 5 miRNA are hsa‐miR‐34a, hsa‐let‐7a, hsa‐miR‐590‐3p, hsa‐miR‐511 and hsa‐miR‐300 and 5 transcription factors are NFYA, USF1, MAX, MYC and TFAP2A. Besides, 58 square blue color node represents miRNA, 22 diamond shape yellow color represents TFs and round shaped maroon 20 seed represent DEGs and black color indicates the 138 edges.

### 3.5 Identification of disease association

**FIGURE 7:**
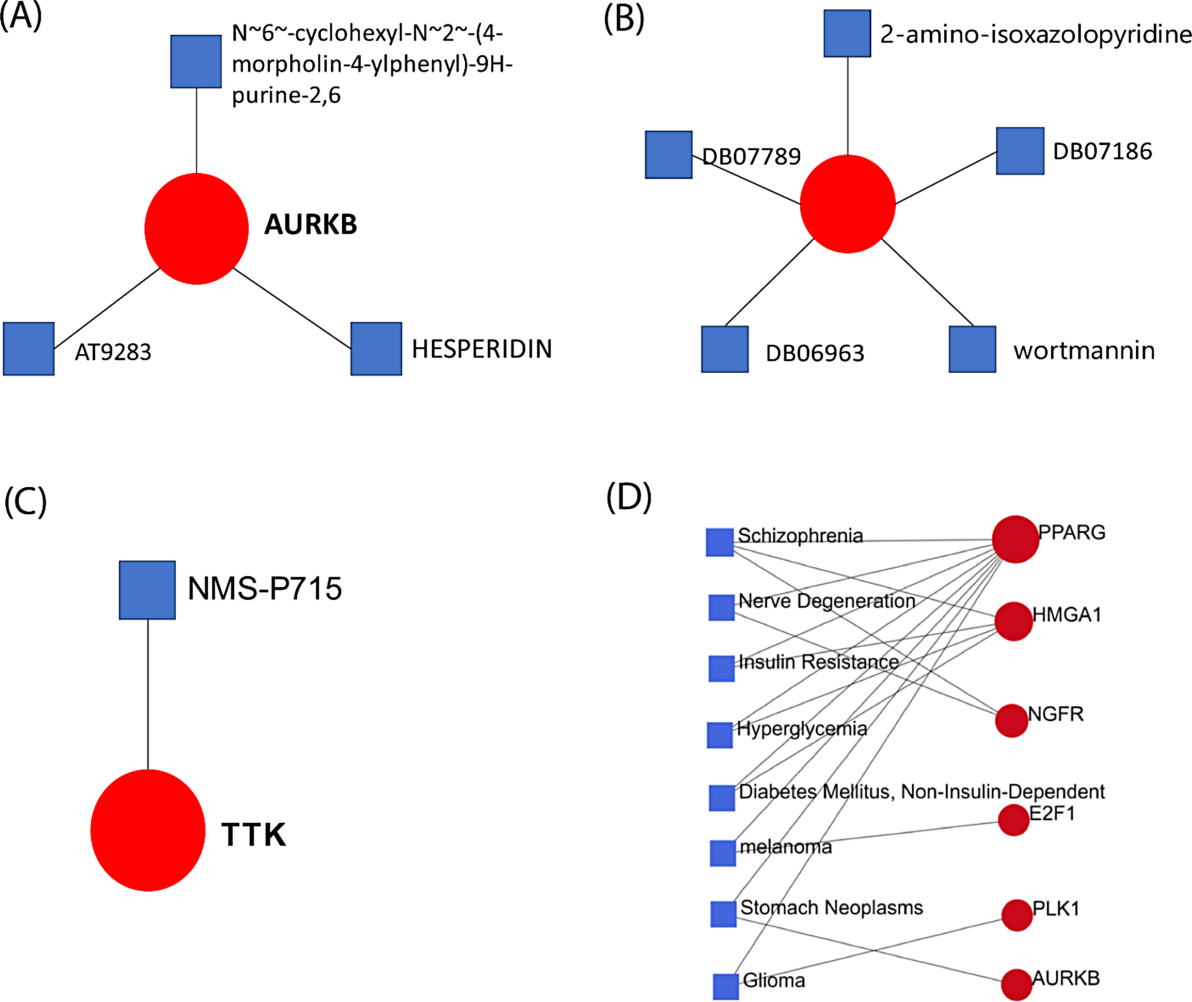
The protein‐drug interaction network analyzed from the (A–C) DrugBank via NetworkAnalyst. Square‐shaped and blue colors represent nine pharmaceutical substances, whereas a circular‐shaped and red color represents the three‐hub gene. (D) Gene‐disease association between identified hub genes with different types of other diseases. Here blue square shape indicates disease name and red round shape indicate our hub gene.

From the NetworkAnalyst inputted gene‐disease association (DisGeNET database), we have shown that 6 possible genes such as PPARG, SMGA1, NGFR, E2F1, PLK1 and AURKB are the most prevalent in Schizophrenia, nerve Degeneration, insulin resistant, hyperglycemia, Diabetes mellitus (non‐insulin dependent), Melanoma, Stomach Neoplasms and Glioma. Besides, PPARG is the most significant gene is responsible for all of these diseases and our shared hub genes AURKB and PLK1 are associated with the disease, respectively, stomach neoplasms and glioma (Figure 7D).

### 3.6 Identification of candidate drugs

The aim was to investigate the protein‐drug interaction and to identify prospective drugs that can potentially change disease processes. Examining protein‐drug interactions is necessary to comprehend the characteristics needed for sensitive receptors. We considered our proposed final 3 hub‐proteins (genomic biomarkers), including AURKB, TTK, and PLK1 showed candidate drugs in protein‐drug interaction in Figure 7A–C.

We found a total 9 candidate drugs from the Drug Bank against AURKB, TTK, and PLK1 proteins, respectively. Among these, we have found 3 drugs for AURKB such as (N~6~‐cyclohexyl‐N ~2~‐(4‐morpholin‐4‐ylphenyl)‐9H‐purine‐2,6‐diamine), AT9283 and hesperidin; only one drug NMS‐P715 for TTK of and five drugs for PLK1, including 2‐amino‐isoxazolopyridine, DB07186 (PubChem ID), DB07789 (PubChem ID), DB06963 (PubChem ID) and Wortmannin.

### 3.7 Drug repurposing through molecular docking simulation

**FIGURE 8:**
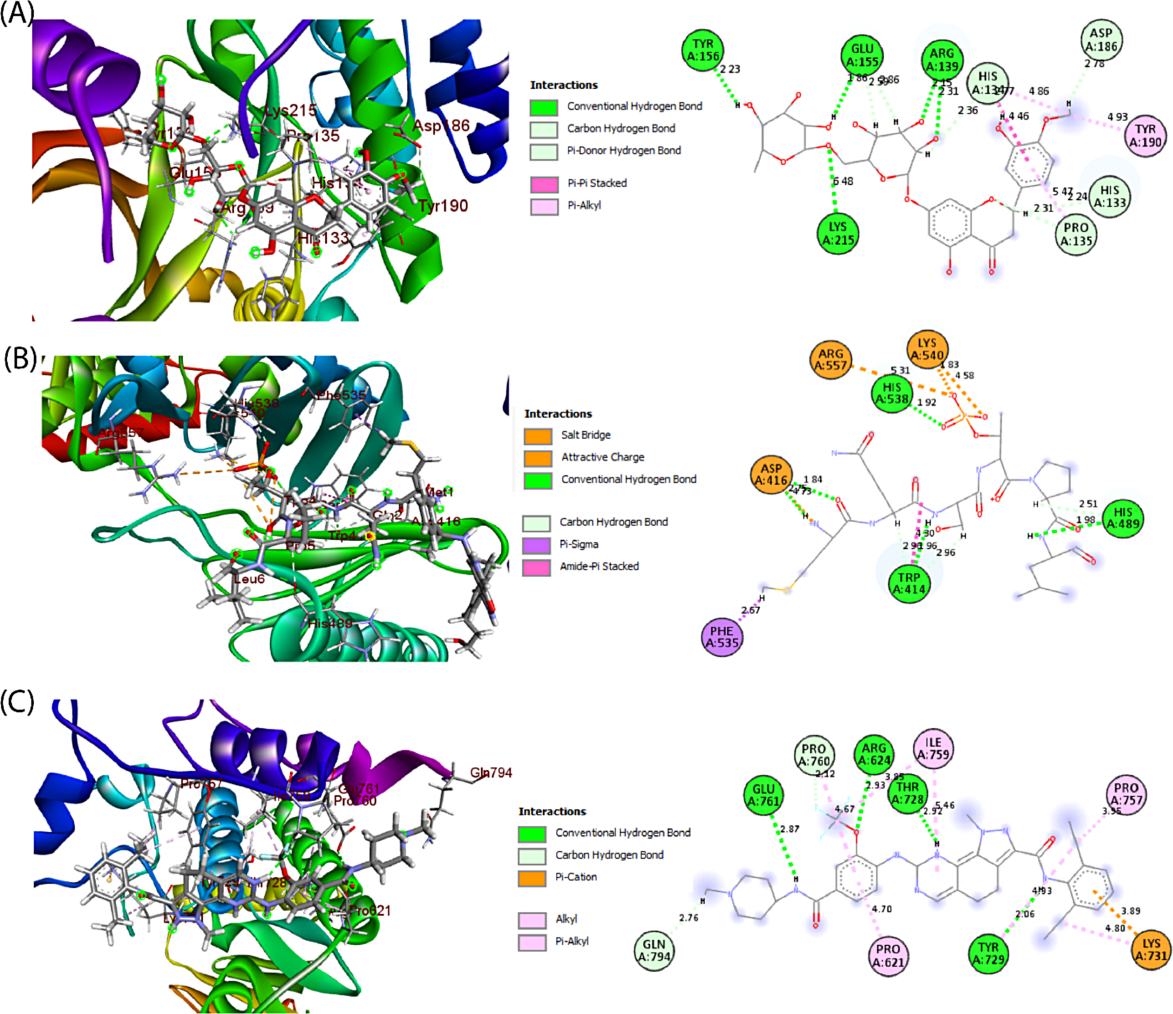
Molecular binding interaction of reported hub proteins and pharmacological agents. (A) represents hesperidin against AURKB protein, (B) 2‐amino‐isoxazolopyridine against TTK protein and (C) NMS‐P715 compound against PLK1 protein.

We used molecular docking modeling to match pharmacological agents with FDA‐approved repurposed medicines that target drug receptors for the treatment of breast cancer. We selected the final hub‐proteins as drug target receptor proteins and proposed pharmacological agents or ligands from DrugBank to conduct molecular docking. From these 3 receptors proteins and total 9 candidate drugs, we took only the top‐ranked 3 lead compounds hesperidin (CID: 10621), 2‐amino‐isoxazolopyridines, (CID: 24941248) and NMS‐P715 (CID:44556162) with significant binding affinity −9.7, − 9.0 and −8.6 (kcal/mol) and their binding amino acids residues were displayed in the Table 2 and Figure 8A–C.

**TABLE 2:** Binding interaction analysis of top 3 lead compounds (drugs) and top 3 probable targets based on Auto‐Dock‐Vina docking results.

Name of potential targets (PDB ID)	Compound ID of Drugs (Control Drugs)	Docking Score of Controls (Kcal/mol)	Docking Score of Drugs (Kcal/mol)	Amino Acids Interaction of Drugs		Number of total bonds
				Hydrogen bond	Hydrophobic bond	
AURKB (4AF3)	CID: 10621 (Tozasertib_CID: 5494449)	−9.5	−9.7	HIS A:133, HIS A:134, PRO A:135, ARG A:139, TYR A:156, and LYS A:215	HIS A:134, PRO A:135, TYR A:190 and SER A:338	13
TTK (2X9E)	CID: 4455612 (Pyrazolo [1,5‐a] pyrimidine_ CID:1163679)	−6.4	−9.0	ARG A:624, THR A:728, TYR A:729, PRO A:760, GLU A:761, and GLN A:794	PRO A:621, TYR A:725, TYR A:729, LYS A: 731, PRO A:757, ILE A:759, and PRO A:760	14
PLK1 (1Q4K)	CID:24941248 (O‐phospho‐L‐threonine_CID:3246323)	−5.3	−8.6	TRP A:416, ASP A:416, HIS A:489, HIS A:538	TRP A:414, ASP A:416, PHE A:535, LYS A:540, and ARG A:557	11

### 3.8 Validation of potential biomarkers

**FIGURE 9:**
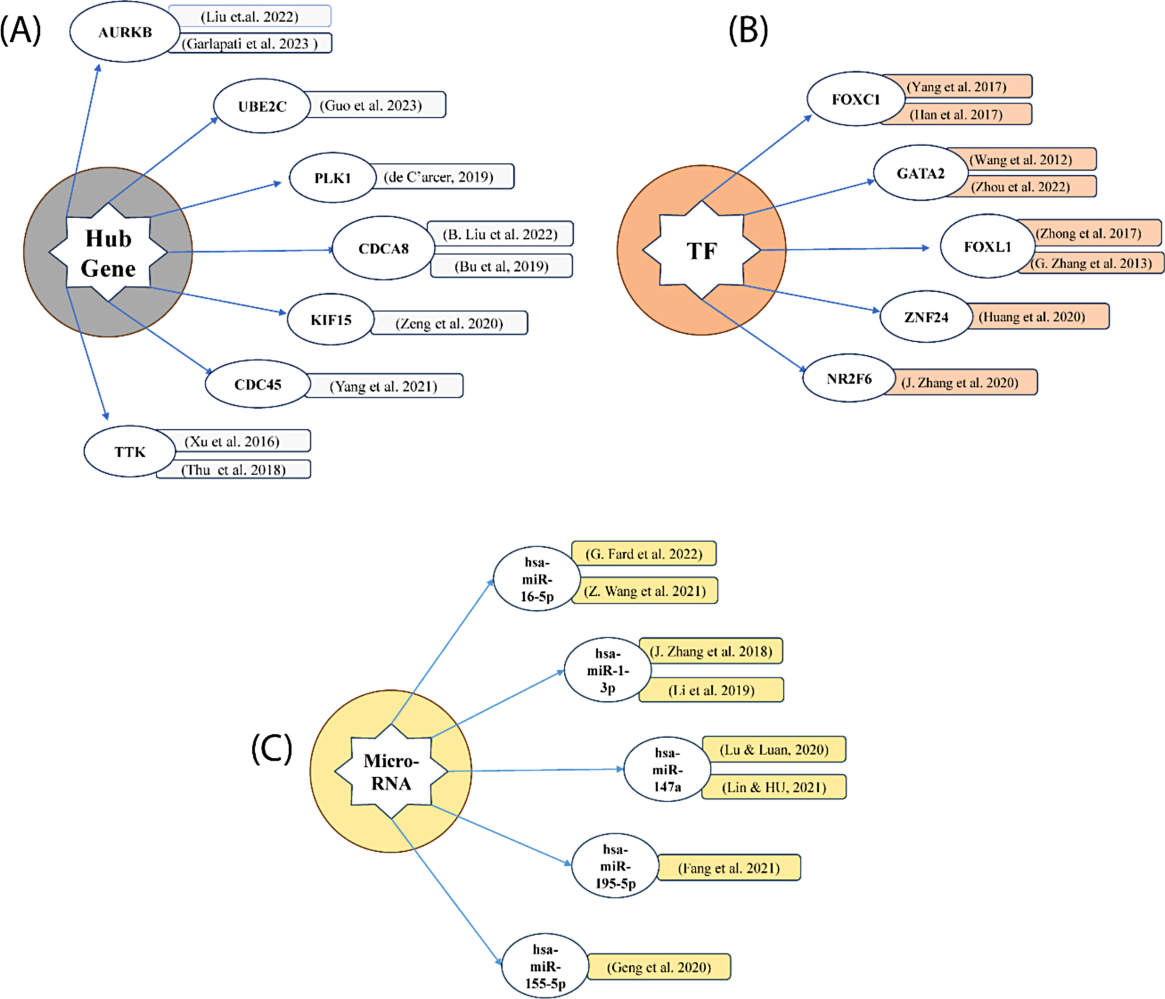
Validation of biomarker through literature analysis. (A) Significant hub proteins, (B) potential micro‐RNAs and (C) transcription factors.

**FIGURE 10:**
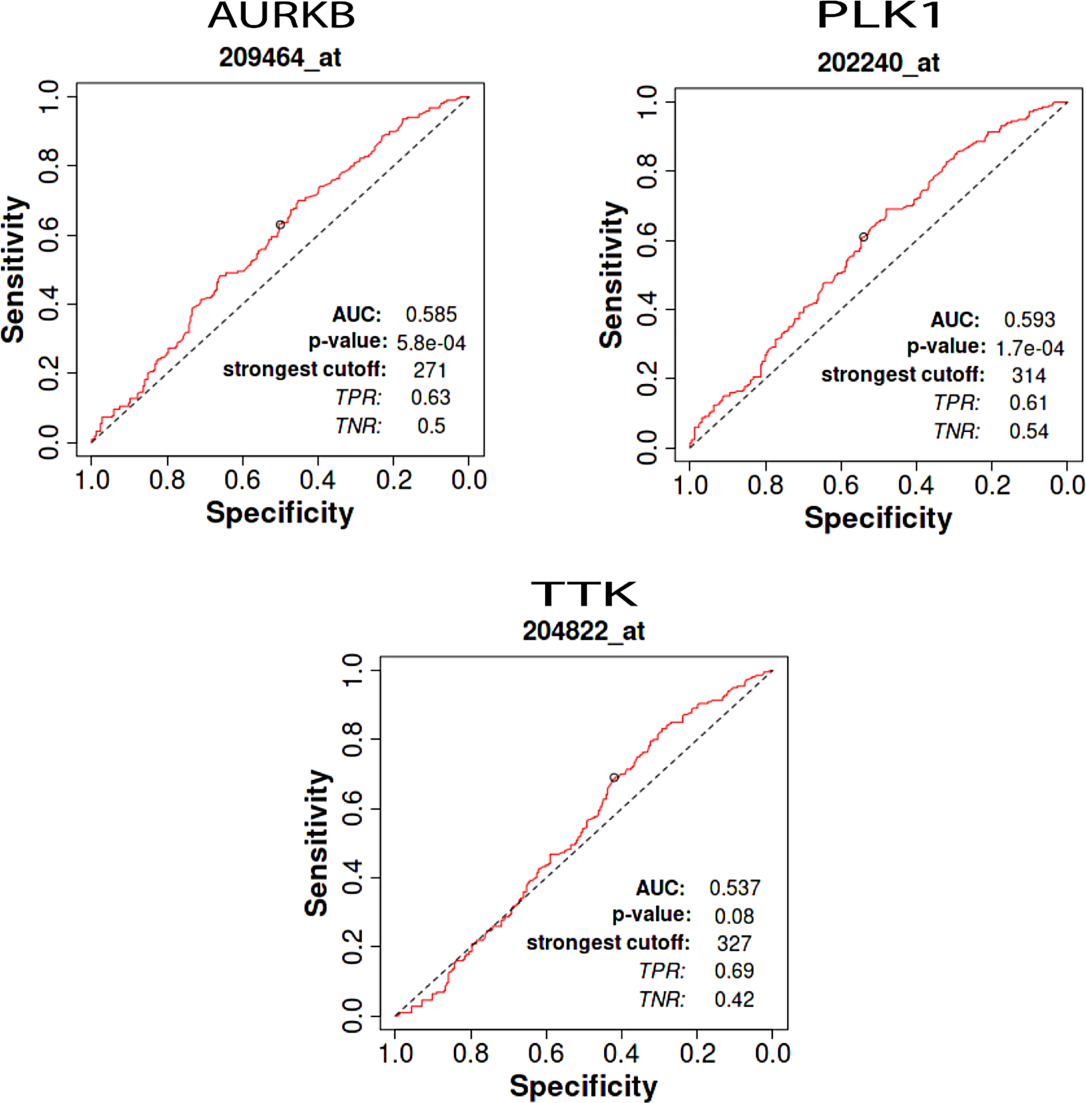
ROC curve analysis to test the validity of gene expression in discriminating tumor and non‐tumor states of the breast cancer samples. The highest total area under the curve (AUC) was found for PLK1 (AUC = 59%, and *p* < .001) and AURKB (AUC = 58%, and *p* < .001), which indicates that PLK1 and AURKB has a good ability to discriminate correctly between tumor and non‐tumor samples.

We identified lots of biomarkers for breast cancer, such as hub proteins, transcription factor (TF) and micro‐RNA (miRNA) that is responsible for the development of breast cancer and we validated the biomarker with a recently published research paper. For Hub protein, we selected 7 genes (AURKB, UBE2C, CDCA8, TTK, KIF15, CDC45 and PLK1) for validation because they are shared by 4 cytoHubba methods (MCC, MNC, Degree and Bottleneck) which are shown in Figure 9A. For Transcription factor (TF), we selected the top 5 such as (FOXC1, GATA2, FOXL1, ZNF24 and NR2F6 from many other transcription factors and we also validated from the previous study that are shown in Figure 9B. For micro RNA (miRNA), we selected the top 5 miRNAs including hsa‐mir‐16‐5p, hsa‐mir‐1‐3p, hsa‐mir‐147a, hsa‐miR195‐5p, and hsa‐miR155‐5p and we also validated the mi‐RNA from previously research study that were shown in Figure 9C. For ROC curve analysis, we have used three proteins including AURKB, PLK1 and TTK as represented in Figure 10. Among them, AURKA (AUC = 0.585 and *p*‐value <.001) and PLK1 (AUC = 0.593 and *p*‐value <.001) showed acceptable values of AUC. On the other hand, TTK (AUC = 0.537 and *p*‐value >.001) demonstrated no discrimination, indicating the capacity to diagnose patients with and without the disease or condition based on the test in our study.

## 4 DISCUSSION

In recent times, bioinformatics breakthroughs have empowered researchers to unveil concealed patterns within intricate biological systems, including those inherent in conditions such as cancer. Hub proteins and regulatory biomarkers may show promise for breast cancer detection and prognosis strategies. In this research, we utilized some bioinformatics analysis for the identification of hub‐proteins as crucial biomarkers for early diagnosis of breast cancer. Besides, we have used drug repurposing approaches to predict some existing drugs for the treatment of breast cancer.

For this study, we have taken four different datasets containing thousands of genes, including up and downregulated genes. Among these four datasets, we found 146 unique common genes shared in every dataset. Then using these unique common genes, we find 12 hub‐proteins through PPI analysis such as AURKB, TTK, PLK1, NUSAP1, UBE2C, ZWINT, CDCA8, CDC25C, KIF15, CDC45, OIP5 and DEPDC1. Among these, three are most significant such as AURKB, TTK, and PLK1 andseveral researchers identified all of these genes have previously been recognized as oncogenes, possible biomarkers for diagnosing and prognosis of early stage BC such as AURKB,^43^ UBE2C,^44^ CDCA8,^45^ CDC45,^46^ KIF15,^47^ TTK^48^ and PLK1.^49^ Besides, the most significant three genes (AURKB, TTK and PLK1) were involved in BC development and served as a potential targets of therapeutic interventions. The overexpression of PLK1 in TNBC patient tissues was validated by Ai Ueda et al. who compared these samples to those of normal mammary glands and benign breast cancers. Finally, the results showed that PLK1 is critical for mitotic regulation in TNBC cells.^50^ Mitosis, spindle formation, and DNA damage response require PLK1 to regulate cell division and genome stability accurately.^51^ Blocking PLK1 expression with antibodies, RNA interference (RNAi), or kinase inhibitors has been found to reduce tumor cell proliferation and induce apoptosis.^52^ In addition, PLK1 and AURKB differentially phosphorylate survivin in order to influence the proliferation of triple‐negative breast cancers that are racially unique.^53^

As a major regulator of the spindle assembly checkpoint (SAC), which works to preserve genomic integrity, TTK has emerged as a viable therapeutic target in human triple‐negative breast cancer (TNBC).^48^ TTK overexpression was considerably greater in basal‐like TNBC and provided a favorable independent predictive biomarker.^54^ Aurora B expression is elevated in breast cancer due to cell proliferation, and co‐deletion of AURKB at 17p13 suggests an integrated system that helps cell clones with impaired mitotic kinase function survive.^55^ Polymorphism of the AURKB gene, as shown by Liao et al. studies may predict disease‐free survival of TNBC patients treated with taxane‐based adjuvant chemotherapy.^56^ Our identified top three hub proteins could serve as biomarkers for the development of early stage breast cancer prognosis and diagnosis confirmed by literature analysis.

In addition, we have used the DAVID database for different pathway analysis, such as gene ontology (GO). Utilizing GO terms and pathways offers a potent approach to comprehend the biological roles embedded within the genes or proteins present in a provided dataset. This methodology aids scientists in understanding the fundamental processes linked to the genes or proteins of concern. Besides, using KEGG pathways holds significant importance in the realms of molecular biology and bioinformatics, offering valuable insights into a range of biological functions, interactions, and processes within organisms.^57^

In the study of Gene Ontology, our identified DEGs are mainly involved in protein binding, ATP binding, transcription factor activation, development of dysfunctional mammary glands, plasma membrane modification, and extracellular exosomes pathways. Blockhuys and Wittung‐Stafshede conducted a study and demonstrated that protein binding plays a role in BC cancer progression and metastasis.^58^ Another study explored that ATP binding contributes to drug resistance in cancer and potentially influences every stage of cancer advancement, including tumor inception, tumor progression, and metastasis.^59^ In addition, transcription factor activation is involved in physiological and developmental processes in tumor and also regulated apoptosis as a molecular function.^60^ In the term of biological process, the development of dysfunctional mammary glands brought on by aging and lipopolysaccharide disrupts milk secretion and aids in BC development.^61^ Cellular components as a plasma membrane modification that regulated resistant drugs, ion channel and lipid bilayer organization.^62^ In addition, exosomes are extracellular vesicles that aided in cellular communication and transcriptional reprogramming of target cells.^63^

However, in the KEGG pathway, we found cytokine‐cytokine receptor interaction, MAPK signaling pathway, and cell cycle etc. Cytokine‐cytokine receptor interaction help vertebrates to BC cell metastasis through intercellular and intracellular communication^64^; p38γ MAPK increased epithelial‐mesenchymal transition (EMT) in BC cells that regulate stem cell of cancer, capacity of self‐renewal and make resistance of target and chemotherapy. Besides, it helps to cancer cell progression and metastasis.^65^ The Reactome pathway is involved in signal transduction, GPCR downstream signaling, and Cell Cycle Checkpoint, etc. One of the fundamental pathways is signal transduction such as the PI3K/Akt/mTOR pathway is involved in survival, growth, proliferation, metabolism, motility and immune response regulation of tumor cells. Mutation makes it tumor cell survival, proliferation and progression, besides antitumor therapies resistant.^66^ Mutation in the cell cycle checkpoint, especially in the S/G1 phase checkpoint, reduced apoptosis of cancer cells and accumulated damaged DNA.^67^ WIKI pathway involves in nuclear receptors meta‐pathway, IL‐18 signaling pathway, and Adipogenesis, etc. Nuclear receptor interactions and crosstalk with other proliferative pathways, such as growth factors helped in the development and treatment of BC.^68^ The adipogenesis pathway plays a crucial role in BC development in several stages. Besides, leptin increase tumor‐associated macrophages (TAMs), such as increasing IL‐18 which activates the NF‐κB/NF‐κB1 signaling pathway that assist migration and invasion of BC cell.^69^

The analysis of gene regulatory networks (GRNs) incorporates both computational and experimental methods.^70^ Computational methods for analyzing gene regulatory networks involve utilizing bioinformatics software such as NetworkAnalyst tools to detect, compare, and study the connections existing between genes and regulatory components.^71^ Employing NetworkAnalyst for protein‐drug interaction, we also predicted possible drugs from the DrugBank that can exhibit efficacy against our biomarker proteins.^72^

In the gene regulatory network by using the NetworkAnalyst web server, we found five (two from Encode and three from Jasper such as FOXC1, GATA2, FOXL1, ZNF24 and NR2F6) potential Transcription Factors (TFs) and all of those are linked with several cancer including breast cancer. FOXC1 is a crucial transcriptional regulator of potential proteins that are associated with carcinomas and regulated genes associated with tumor. Abnormal expression of FOXC1 is involved in maintaining cancer stem cell proliferation, migration and angiogenesis.^73^ According to Wang et al. studies, overexpressed GATA2 caused human breast carcinomas by blocking PTEN, which promoted the growth and stimulation of BC.^74^ Besides, it mutated TP53 which help to survive cancer cell by the Notch signaling pathway.^75^ Overexpression of FOXL1 slows down β‐catenin, c‐Myc, and cyclin D1 expression, inhibiting breast cancer cell invasion and migration.^76^ Research shows that upregulated ZNF24 increases tumor volume, migration and invasion through EMT process.^77^ Another research show that NR2F6 is vital for immune surveillance in cancer and poor chemotherapy survival.^78^

Besides, we also used NetworkAnalystto predict our effective microRNA (miRNA) that play an essential role in BC as well as other cancers. We found top five miRNAs such as hsa‐miR‐16‐5p, hsa‐miR‐1‐3p, hsa‐miR‐147a, hsa‐miR‐195‐5p, and hsa‐miR‐155‐5p from miRTarbase and Tarbase interaction. Study shows that hsa‐miR‐16‐5p play a role in carcinogenesis and help to malignancies such as osteosarcoma, cervical cancer, brain tumors, breast cancer, bladder cancer and lung cancer^79^ and overexpression help to block G2/M phase that increases apoptosis in BC cells.^80^ Targeting glutaminase 3’‐UTR with hsa‐miR‐1‐3p and overexpressing it reduces lung adenocarcinoma cell viability and invasion.^81^ Lu and Luan concluded that, decrease growth and metastasis of non‐small‐cell lung cancer effects on upregulated hsa‐miR‐147a,^82^ while dysregulated hsa‐miR‐147a responsible for many diseases such as cancer, infectious, and cardiovascular disease.^83^ On the other hand, hsa‐miR‐195‐5p is responsible for NUAK2 gene expression level alteration and plays a crucial role in tumor progression.^84^ Besides, hsa‐miR‐155‐5p plays a role in carcinoma development and acts as an apoptosis factor.^85^

Therefore, in this research, we identified the top three potential drugs that can bind with our leading hub protein and regulate it. Hesperidin (CID: 10621) is aurora kinase inhibitor specific, as evidenced by reduced histone H3 phosphorylation and a phenotype comparable to AURKB knockdown.^86^ Hanan et al. conducted a study by designing and synthesizing 2‐amino‐isoxazolopyridines (CID: 24941248) as Polo‐like kinase inhibitor.^87^ The particular suppression of TTK activity by the NMS‐P715 (CID: 44556162) molecule is associated with potential anti‐proliferative action in human cancer cells, as seen in both in vitro and in vivo experiments using mouse xenograft models.^88^ Consequently, the proposed candidate drugs may be essential in treating BC by targeting respected three hub proteins with other drug efficacy testing.

Although there are some limitations in our work, including the lower number of datasets with small sample size, despite available datasets in GEO and TCGA databases, statistical error during some data procession or normalization, different tissue sources and only a few statistical operations were employed. Finally, we identified some potential hub proteins, regulatory biomarkers and also predicted existing drugs that have already been studied by many researchers. In the future, we would like to conduct our work on wet‐lab validation using animal trials that will give us a chance to properly implement our work and may help to start a cutting‐edge process of computational and wet‐lab research.

## 5 CONCLUSIONS

The objective of the study was to use bioinformatics analysis to discover and rule out key hub proteins and regulatory biomarkers linked to early detection of breast cancer. Using an integrated strategy, different computational techniques were used to examine complicated biological data and identify important proteins and indicators that are crucial to the initiation and progression of breast cancer. By construction of protein–protein interaction (PPI) network of 146 DEGs, we found three hub proteins (AURKB, TTK and PLK1) by employing five different cytoHubba methods. Several other research that we listed in the discussion section also reported their link with BC, either directly or indirectly. Our identified some crucial GO terms of each (BP, MF, and CC) and signaling pathways (KEGG, WiKi and Reactome) were considerably enriched by DEGs, including three hub proteins. Key pathogenesis pathways in BC progression were determined to be the enriched GO keywords and signaling pathways, which were corroborated by a review of the relevant literature. We detected five transcription factors such as FOXC1, GATA2, FOXL1, ZNF24, and NR2F6 and five micro‐RNA such as hsa‐miR‐16‐5p, hsa‐miR‐1‐3p, hsa‐miR‐147a, hsa‐miR‐195‐5p, and hsa‐miR‐155‐5p, were also identified as the key transcriptional and post‐transcriptional regulators of hub proteins. These regulatory factors significantly influence the regulation of key hub proteins. These all findings are strongly connected with the development and progression of Breast Cancer. Finally, we have predicted some drugs (Hesperidin, 2‐amino‐isoxazolopyridines, and NMS‐P715 as inhibitors against three common hub protein genes through molecular docking simulation. This study not only advances our knowledge of the molecular processes that give rise to breast cancer, but it also offers possible channels for early diagnosis and specialized treatment plans. Before using these findings in clinical trials, more molecular analysis, including in‐vivo and in‐vitro studies is required.

## AUTHOR CONTRIBUTIONS

**Most Shornale Akter**: Conceptualization; formal analysis; methodology; writing‐original draft. **Md. Helal Uddin**: Data curation; formal analysis; investigation; methodology; resources. **Sheikh Atikur Rahman**: Conceptualization; data curation; formal analysis; software; visualization. **Md. Arju Hossain**: Conceptualization; formal analysis; methodology; writing‐review and editing. **Md. Ashiqur Rahman Ashik**: Data curation; formal analysis; software. **Nurun Nesa Zaman**: Formal analysis; investigation; validation. **Omar Faruk**: Investigation. **Md. Sanwar Hossain**: Writing‐review and editing. **Anzana Parvin**: Conceptualization; investigation, project administration, supervision. **Md Habibur Rahman**: Conceptualization; project administration; supervision; writing‐original draft; writing‐review and editing.

## CONFLICT OF INTEREST STATEMENT

The authors have stated explicitly that there are no conflicts of interest in connection with this article.

## ETHICS STATEMENT

None.

## Data Availability

Data included in articles will be provided upon on author request.
